# Purification, characterization, and determination of biological activities of water-soluble polysaccharides from *Mahonia bealei*

**DOI:** 10.1038/s41598-022-11661-3

**Published:** 2022-05-17

**Authors:** Mohib Ullah Kakar, Jingyi Li, Muhammad Zubair Mehboob, Rokayya Sami, Nada Benajiba, Aziz Ahmed, Amina Nazir, Yulin Deng, Bo Li, Rongji Dai

**Affiliations:** 1 Beijing Key Laboratory for Separation and Analysis in Biomedicine and Pharmaceutical, School of Life Sciences, Beijing Institute of Technology (BIT), Beijing, 100081 China; 2 Faculty of Marine Sciences, Lasbela University of Agriculture, Water and Marine Sciences, (LUAWMS), Uthal, Balochistan Pakistan; 3 CAS Centre for Excellence in Biotic Interaction, College of Life Sciences, University of Chinese Academy of Science, Beijing, 100049 China; 4 Department of Food Science and Nutrition, College of Sciences, Taif University, P.O. 11099, Taif, 21944 Saudi Arabia; 5 Department of Basic Health Sciences, Deanship of Preparatory Year, Princess Nourah bint Abdulrahman University, P.O. Box 84428, Riyadh, 11671 Saudi Arabia; 6 Institute of Animal Science and Veterinary Medicine, Shandong Academy of Agricultural Sciences, Jinan Industry North Road 202, Jinan, Shandong Province China; 7 Advanced Research Institute of Multidisciplinary Sciences, Beijing Institute of Technology, Beijing, 100081 China

**Keywords:** Psychology, Chemistry

## Abstract

*Mahonia bealei* is one of the important members of the genus Mahonia and Traditional Chinese Medicine (TCM). Several compounds isolated from this plant have exhibited useful biological activities. Polysaccharides, an important biomacromolecule have been underexplored in case of *M. bealei*. In this study, hot water extraction and ethanol precipitation were used for the extraction of polysaccharides from the stem of *M. bealei*, and then extract was purified using ultrafiltration membrane at 50,000 Da cut off value. Characterization of the purified *M. bealei* polysaccharide (MBP) was performed using Fourier Transform Infrared Spectroscopy (FT-IR), along with Scanning Electron Microscopy (SEM), X-ray crystallography XRD analysis and Thermal gravimetric analysis (TGA). The purified polysaccharide MBP was tested for antioxidant potential by determining its reducing power, besides determining the DPPH, ABTS, superoxide radical, and hydroxyl radical scavenging along with ferrous ion chelating activities. An increased antioxidant activity of the polysaccharide was reported with increase in concentration (0.5 to 5 mg/ml) for all the parameters. Antimicrobial potential was determined against gram positive and gram-negative bacteria. 20 µg/ml MBP was found appropriate with 12 h incubation period against *Escherichia coli *and *Bacillus subtilis* bacteria. We conclude that polysaccharides from *M. bealei* possess potential ability of biological importance; however, more studies are required for elucidation of their structure and useful activities.

## Introduction

*Mahonia* is a genus belonging to basal eudicots. It consists of small trees and shrubs that are evergreen. These plants possess compound leaves which are coriaceous (sclerophyllous)^1,2^. Geographically this plants is distributed throughout the globe and at least twenty species have been found in some southwestern parts of the United States^2^ as well as Europe, where members of this genus grow as invasive species^3^. *Mahonia bealei* is an important member of this genus which bears medicinal importance.

Traditional Chinese Medicine (TCM) uses different materials which are derived from animals, plants as well as minerals and it has a long history. Different diseases like dysentery have been treated since long by using different parts of Mahonia, including roots, stem, and leaves as an important part of the TCM. Important features of treatment of this plants include its moisturizing effect, as a detoxifying agent and having property of clearing heat (Chinese Pharmacopeia Commission 2010)^4^.

At least ten or more monosaccharide units combine through glycosidic bonds to form complex polysaccharides. All the living cells contain polysaccharides; however, in plants they perform important functions. Plant polysaccharides have been explored throughout the history for performing useful activities which include antiaging, anticancer and immunoregulatory activities. Due to successful lowering of blood lipid levels these polysaccharides have been successful in treating cardiovascular disorders. Additionally, these molecules are reported to successfully lower the blood glucose levels and treat diabetes^5–13^. These polysaccharide molecules play an important role in developing food products, packaging materials as well as therapeutic products^14–18^.

Antioxidants are another important type of compounds that inhibit the oxidation process and help in the reduction of free radicals. This property helps in relieving oxidative stress from the body upon their use. The long term use of synthetic antioxidant agents have reported to cause carcinogenesis as well as liver injury^19^. Polysaccharides from different plants have exhibited potential antioxidant activities and we have already reviewed some of them previously^17,18,20^. Polysaccharides from *M. bealei* and their antioxidant potential has remained unexplored according to our survey of literature.

*Mahonia caulis* is an important member of the TCM. It is comprised of *Mahonia bealei* (Fort.) Carr. Or *M. fortune* (Lindl.) Fedde dried stems and is one of the important medicines used in the TCM. Different diseases have been treated using it which include ulcers, carbuncles, icteric hepatitis conjunctivitis, toothache, boils and other diseases that have been related to stomach fire^21^. Leaves of *Mahonia bealei* have been reported as rich in polyphenolic compounds and possess antioxidant activities, these have been used for the production of bitter tea^22^. Numerous studies show the exhibition of antioxidant potential through consumption of leaves of plants. These are used in the form of tea, for example, black tea used worldwide is a classic example of this potential^23^. Stem and roots of this plant contain alkaloids and cerebrosides according to phytochemical studies^24^. Traditionally, in the TCM, certain parts of plant species belonging to genus Mahonia including fruits, bark, stem, leaves, and roots have been used for therapeutic purposes^25–29^. Alkaloids, sterols, glycosides, and flavonoids have been isolated from *M. bealei*^30^. Several studies have reported that compounds extracted and purified from different parts of *M. bealei* possess useful biological activities like antioxidant activity^22,31^, antimicrobial activity^27,32–35^, anti-inflammatory^36^, anti-gastrin^37^, as well as antitumor activities^22,38–40^.

Besides several studies conducted on extraction and purification of compounds from *M. bealei*, the extraction and purification aspect of polysaccharides, as well as exploration of their useful therapeutic potential has been underexplored according to our knowledge. Previously, we reviewed phytochemistry and useful biological activities of compounds isolated from *M. bealei* and their medicinal importance^41^. In the current study, we extracted polysaccharides for the stem of *M. bealei*, purified, and characterized them besides exploring their useful biological activities.

## Materials and methods

This study was conducted using the following scheme of experiments.

### Collection of the plant for this study

The plant *M. bealei* was collected from the Yunnan Province of China in 2018. It was identified by Professor Rongji Dai of the Beijing Key Laboratory for Separation and Analysis in Biomedicine and Pharmaceuticals, School of Life Science, Beijing Institute of Technology (Beijing City, China) and it was stored in herbarium with specimen number 1850 at the Beijing Institute of Technology, (BIT), Beijing. The whole *M. bealei* plant samples were dried before processing and were in accordance with the China's primary agricultural product standards. Identification was based on Pharmacopoeia of the Peoples Republic of China 2015.

### Extraction of polysaccharides by water extraction method

*Mahonia bealei* stem weighing about one kg was grounded, powdered, and mixed for extraction. Then hot water was used for extraction at a ratio of 1:10 w/v (material to solvent). Distilled water was used, and this material was boiled to 100 °C for one hour and the whole process was repeated four times. Every time mixture was cooled and filtered to collect the supernatant.

This supernatant was treated with 75% ethanol (1:4, extract to ethanol ratio) and kept at 4 °C for 24 h. A pellet was formed containing polysaccharides and protein which was further separated. Centrifugation for this mixture was performed for ten minutes at 4000 rpm. The resultant pellet contained the desired extract which was further processed.

Further filtration was performed to separate ethanol using 0.45 µ filter paper (Whatmann). Then water soluble parts of the sample were collected using rotary evaporator and were then concentrated as well as dried in a dry oven for complete evaporation of water content. A completely dry sample was collected and analyzed further.

Deionized water was used to dissolve the freeze-dried extract. 10 mg/ml concentration was used for the dissolution. Sevage reagent (n-butanole-chloroform, 1:4) was used for final separation of proteins from the mixture at a concentration of 1/4^42^.

### Ultrafiltration using membrane for removal of small molecules

**Figure 1:**
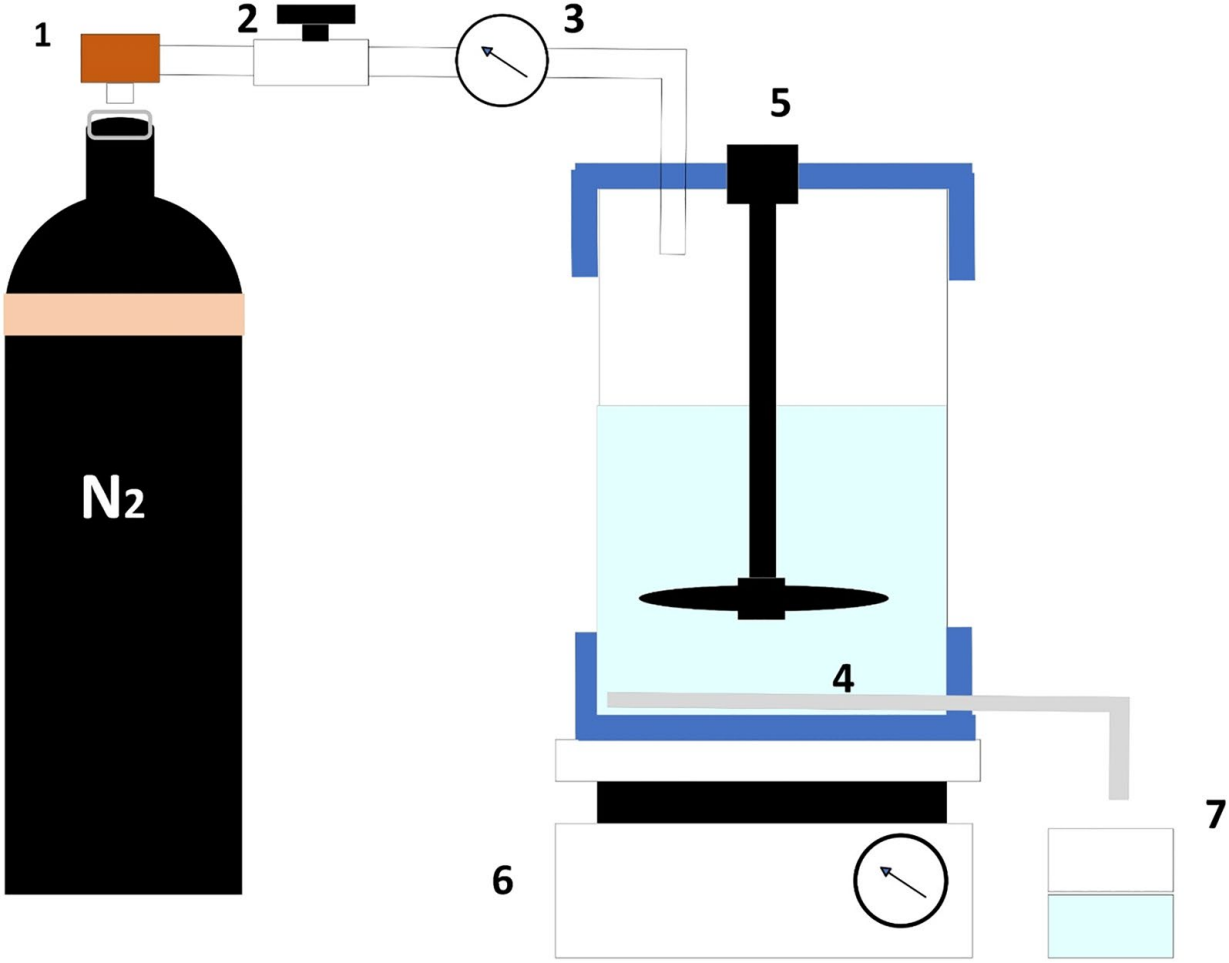
Ultrafiltration setup: 1—Tank (containing nitrogen), 2—Regulator (for pressure), 3—Manometer, 4—Membrane, 5—Stirred ultrafiltration cell, 6—Magnetic stirrer, 7—Container (for permeate), Figure adopted from^44^.

For ultrafiltration, completely dried samples were dissolved in water until complete dissolution. This sample was passed through the membrane (50,000 Da, cut-off for MW) and was intensively dialysed so that other molecules are removed from this dissolved sample. These included compounds like flavonoids, polyphenols etc. The sample obtained was further concentrated, dried and processed^43^. The process of ultrafiltration is shown in the Fig. 1 below:

### Characterization of *M. bealei* polysaccharides (MBP)

After extraction and purification of polysaccharides, MBP were characterized using different methods. These are discussed below.

#### FT-IR spectroscopy

This analysis was performed by using a FT-IR spectrophotometer (FT-IR, Nicolet, USA) model 5700. An OMNIC workstation operating in a 4000–400 cm^−1^ range was used^45^. 1 mg sample of polysaccharide and 50 mg KBr were mixed. Agate pestle and mortar was used for grounding of the sample, and it was further analyzed after pressing in pellets.

#### Scanning electron microscopy (SEM)

For SEM, field emission SEM, (JEOL Ltd. Japan), model JSM 670IF was used. Sample was freeze dried before attachment with the adhesive tape that was double sided. It was coated with platinum for analysis after attachment with the specimen stub. Finally, the images obtained by SEM of the MBP were recorded.

#### XRD analysis

XRD analysis was performed for our extracted samples according to the previous studies^46^. For analysis, X-ray diffractometer, (D8 ADVANCE, Bruker, Germany) was used and the process was performed at room temperature, that is, 20 ± 1 °C. 5°–50° of 2*θ* range was used for the collection of patterns. 0.02° step size was applied at a speed of 1 s/step for this purpose.

#### Thermal gravimetric analysis (TGA)

This analysis was performed in accordance with previous studies^47^. The instrument used for this purpose was TGA4000, (PE Corporation, USA). TGA microbalance was used for weighing 2–5 mg of freeze-dried sample and it was heated at 20 °C/min rate from 30 to 600 °C. For heating, 20 mL/min flow rate was applied using nitrogen gas.

### Estimation of biological activities from *Mahonia bealei* polysaccharides

Different biological activities exhibited by MBP were analysed in the next part of study. A detail of methods followed for this study are as follows.

#### Antioxidant activity

Among several useful biological activities, antioxidant activity is among the most important ones. It was evaluated using various methods described in detail as under:

#### Determination of reducing power of MBP

MBP sample was analysed for determining its reducing potential using previously described methods; however, slight modifications were made^48^. I ml of MBP polysaccharide sample was prepared and then mixed using phosphate buffer saline (0.2 M) at 6.6 pH and 2.5 mL volume. Different range of sample concentrations was prepared from 0, 0.5, 1.0, 1.5, 2, 2.50, 3, 3.5, 4 and 5 mg/ml. 2.5 mL potassium ferricyanide K_3_Fe(CN)_6_ (1% (w/v) solution was prepared. After mixing with this mentioned solution, sample was incubated for 20 min at 50 °C. Then 2.5 ml TCA solution (10% w/v) was added. The mixture was subjected to centrifugation for 10 min at 1500×*g* or 4200 rpm. Then upper layer (2 ml) was picked. It was mixed with the same amount of distilled water before adding 0.1% (w/v) FeCl_3_. The resulting solution was analysed using spectrophotometer and absorbance was checked at 700 nm taking Vitamin C as a positive control. This control is used in this experiment as Vitamin C is a natural oxidant. An increased amount of absorbance was considered displaying a greater reducing power.

#### Measurement of DPPH free radical scavenging activity

This parameter was used for determining the antioxidant activity of MBP according to the reported studies after slight modifications^49^. 3 mL DPPH was prepared. This reagent as a source of free radicals was prepared using 0.1 mM concentration in 50% ethanol. 1 ml of sample concentration was prepared in a range of different concentrations 0, 0.5, 1.0, 1.5, 2, 2.50, 3, 3.5, 4 and 5 mg/ml. The prepared mixture was incubated for 25 min at 25 °C. It was vigorously shaked for 5 min. The absorbance at 517 nm was measured for this mixture and Vitamin C was applied as a positive control. Ascorbic acid is a natural antioxidant which is applied here as a positive control. Free radical scavenging activity was then measured using the above readings and following formula:

$$ {\text{Scavenging }}\;{\text{activity }}\left( \% \right) \, = \, \left( {{\text{A}}_{0} - {\text{ A}}_{{1}} /{\text{ A}}_{0} } \right) \, \times { 1}00 $$

Here, A_0_ corresponds to the absorbance (at 517 nm) of the control. Control solution contained DPPH and no sample was added. A_1_ corresponds to sample absorbance (517 nm). Sample means the positive control or DPPH solution mixed with the sample.

#### Measurement of ABTS radical scavenging activity

ABTS free radicals were applied to determine the free radical scavenging activity of MBP polysaccharides as reported previously after some modifications. 7 mM ABTS solution and 2.45 mM potassium persulfate solutions were mixed. The reaction was carried out in the dark for 12–16 h. Phosphate buffer (pH 7.4) was used for diluting the ABTS solution to 50–60 times and its absorbance was adjusted to 0.70 ± 0.02 at 734 nm wavelength. Sample solution (0.4 ml) was prepared at different concentrations that ranged from 0, 0.5, 1.0, 1.5, 2, 2.50, 3, 3.5, 4 and 5 mg/ml. The mixture was added to 3 ml of ABTS solution. Spectrophotometer was used for determining the absorbance at 734 nm before vigorous mixing, and it was stabilized by keeping at room temperature within 10 min of measurement. Ascorbic acid was the positive control. This was applied being a natural antioxidant and the following formula was applied for calculating the ABTS radical scavenging ability;

$$ {\text{Scavenging }}\;{\text{activity }}\left( \% \right) \, = \, \left( {{\text{A}}_{0} - {\text{ A}}_{{1}} /{\text{ A}}_{0} } \right) \, \times { 1}00 $$

Here, A_0_ represents the control (absorbance). ABTS solution, lacking any sample was the control. A_1_ shows the reading (absorbance) of sample (MBP sample along with ABTS) or the positive control.

#### Superoxide radical scavenging activity

Antioxidant ability of MBP was tested through the use of superoxide anion radical after modifying the previously reported protocol^50^. For this test 0.5 ml of MBP was prepared at different concentration range from 0, 0.5, 1.0, 1.5, 2, 2.50, 3, 3.5, 4 and 5 mg/ml. Tris–HCl buffer (50 mM, pH 8.2) was used in 5 ml volume for mixing. Further the mixture was vigorously shaken after adding 0.5 ml of pyrogallic acid (5 mM) solution. At 25 °C the mixture was incubated for ten minutes, and it was terminated by dripping 0.1 ml of HCl (0.1 M). In this experiment Vitamin C was used a positive control and distilled water was applied as a blank. Vitamin C is a natural antioxidant, so it was used as a positive control for comparison and distilled water was applied as a blank in this experiment. 420 nm wavelength was set for measuring the absorbance of this mixture. Later, the following formula was used for the determination of scavenging ability of MBP of the superoxide:

$$ {\text{Scavenging}}\;{\text{ activity }}\left( \% \right) \, = \, \left( {{\text{A}}_{0} - {\text{ A}}_{{1}} /{\text{ A}}_{0} } \right) \, \times { 1}00 $$

Here A_0_ represents the absorbance of blank (deionized water) and A_1_ represents the absorbance of the MBP sample.

#### Hydroxyl radical scavenging ability

Antioxidant ability of the MBP was analysed by using the hydroxyl radical scavenging potential of the MBP as mentioned previously^51^. A concentration ranges from 0, 0.5, 1.0, 1.5, 2, 2.50, 3, 3.5, 4 and 5 mg/ml. of 1 ml polysaccharide was prepared. 1 ml of salicylic acid–ethanol (9 mmol/l concentration) and 1 ml ferrous sulfate (9 mmol/l concentration) were used for mixing the sample before start of the reaction. Hydrogen peroxide at 8 mmol/l concentration and 1 ml volume was used to start the reaction. Then spectrophotometer was used to record the absorbance at 510 nm and the mixture was incubated at 37 °C for 30 min. In this experiment distilled water (1 ml) was used as a blank and Vitamin C (ascorbic acid, a natural antioxidant) was used as a positive control.

The activity was calculated by the following formula,

$$ {\text{Scavenging }}\;{\text{activity }}\left( \% \right) \, = \, \left( {{\text{A}}_{0} - {\text{ A}}_{{1}} /{\text{ A}}_{0} } \right) \, \times { 1}00 $$

Here A_0_ represents the absorbance of blank (distilled water) and A_1_ shows the absorbance of sample.

#### Determination of ferrous ion chelating activity

For this experiment, iron-ferrozine complex was used and ferrous ion chelating activity was estimated for MBP sample depending upon the decrease in absorbance according to the previous study^52^. MBP sample was prepared in 1 mlvolume and different range of concentration, that is, 0, 0.25, 0.5, 0.75, 1.0, 1.5, 2, 2.50, 3, 3.5, 4 and 5 mg/ml and it was mixed with 100 µl quantity of FeCl_2_·4H_2_O (2.0 mmol/l). Distilled water (3.7 ml) was also added to the mixture. Ferrozine (5.0 mmol/l) was used to start the reaction. For this purpose, 200 µl ferrozine was added. The mixture was left for 20 min to attain equilibrium. Then absorbance of the reaction mixture was recorded at 562 nm wavelength. In this experiment EDTA was used as positive control as studies have shown that EDTA exhibits ferrous ion chelating activity; therefore, it has been applied as the positive control in this study. Following formula was used for the calculation,

$$ {\text{Scavenging }}\;{\text{activity }}\left( \% \right) \, = \, \left( {{\text{A}}_{0} - {\text{ A}}_{{1}} /{\text{ A}}_{0} } \right) \, \times { 1}00 $$

Here A_0_ shows the absorbance of blank (distilled water) and A_1_ shows the absorbance of the sample.

### Estimation of antibacterial activities of MBP

The polysaccharide sample obtained from *Mahonia bealei* stem was tested for its antibacterial potential. Following steps were used to analyze this potential.

#### Preparation of sample

Antibacterial activity of the extracted, purified and characterized polysaccharides obtained from the stem of *Mahonia bealei* was tested. Different concentrations of this confirmed compound were made after dissolving in water. A range of concentrations of the MBP sample was prepared from 5, 10, 15, 20, and 25 µg/ml. These concentrations were dissolved in 1 ml of water for sample preparation. These different concentrations were used for testing as potential antibacterial agents.

#### Media preparation and bacterial culture

*Bacillus subtilis* and *Escherichia coli* cultures were tested in this study. These were obtained after normal growth in the laboratory. Luria Broth (LB) medium was used as a culture media for the growth of these microorganisms and determination of their antibacterial potential. Composition of LB per liter included, peptone 10 g, sodium chloride 5 g, yeast extract 5 g, and agar 15 g dissolved in distilled water (1 l).

This media was sterilized by autoclaving at 121 °C under pressure for 15 min. It was then poured aseptically in the petri plates in laminar flow hood to avoid any contamination. Phosphate buffer saline (PBS) was also prepared for the experiment. A stock solution was prepared using KCl 2.7 mM, NaCl 137 mM, KH_2_PO_4_ 1.8 mM, and Na_2_HPO_4_ 10 mM. Finally, pH of the PBS was adjusted to 7.2. Hydrochloric acid HCl and sodium hydroxide NaOH were used for pH adjustment.

#### Preparation of microbial culture

*Bacillus subtilis* was used as a model organism for representing Gram (+) bacteria, and *E. coli* was used as a model organism for testing antibacterial potential of MBP against Gram (−) bacteria. Bacterial cultures were grown overnight at 37 °C. A colony of pure culture was transferred to flask containing LB broth. After growth of culture to log phase, bacterial population was pelleted after centrifuging culture broth (1 ml) at 8000 rpm for 10 min. PBS was used to wash the pellet. It was then redissolved in the PBS for preparing cell suspensions. A final concentration of 10^7^ cells per mL was used for preparing the sample. This final concentration of bacterial cells was used for testing the efficiency of different concentrations of MBP against bacterial growth.

#### Antibiotic susceptibility testing

For measuring the antibacterial efficacy, colony count of bacterial cells and optical density (OD) were measured. Antibacterial activity was determined using batch assays for determining the efficiency of different concentrations of the compound.

Petri plates containing LB media were prepared, and bacterial culture was distributed on all the plates. Different range of concentration of MBP was used, that is, 0–20 µg/ml on petri plates. The bacteria were spread evenly on the plates before the application of compound. After pouring the MBP sample, the inoculated peri plates were incubated at 37 °C using incubator for 24 h. The growth of bacteria on petri plates was observed to check the inhibition ability of the different concentrations of the polysaccharide. Optical density (OD) was tested to measure the inhibitory concentration of the compound against both these bacteria. Cultures were grown in separate LB flasks by continuous shaking at 150 rpm and 35 °C for 4 h. DI was used as a control. Tubes (15 ml) containing LB medium (10 ml) were inoculated and then kept in an orbital shaking incubator at 35 °C and 150 rpm.

Optical density (OD) of the samples was calculated at 600 nm wavelength in all the aliquots containing the samples. Readings were taken at specific time intervals. The growth of bacteria measured according to the corresponding OD values was recorded and data was plotted to determine bacterial growth inhibition. For precision, experiments were performed in triplicate and for final results average of all the readings was taken.

**Figure 2:**
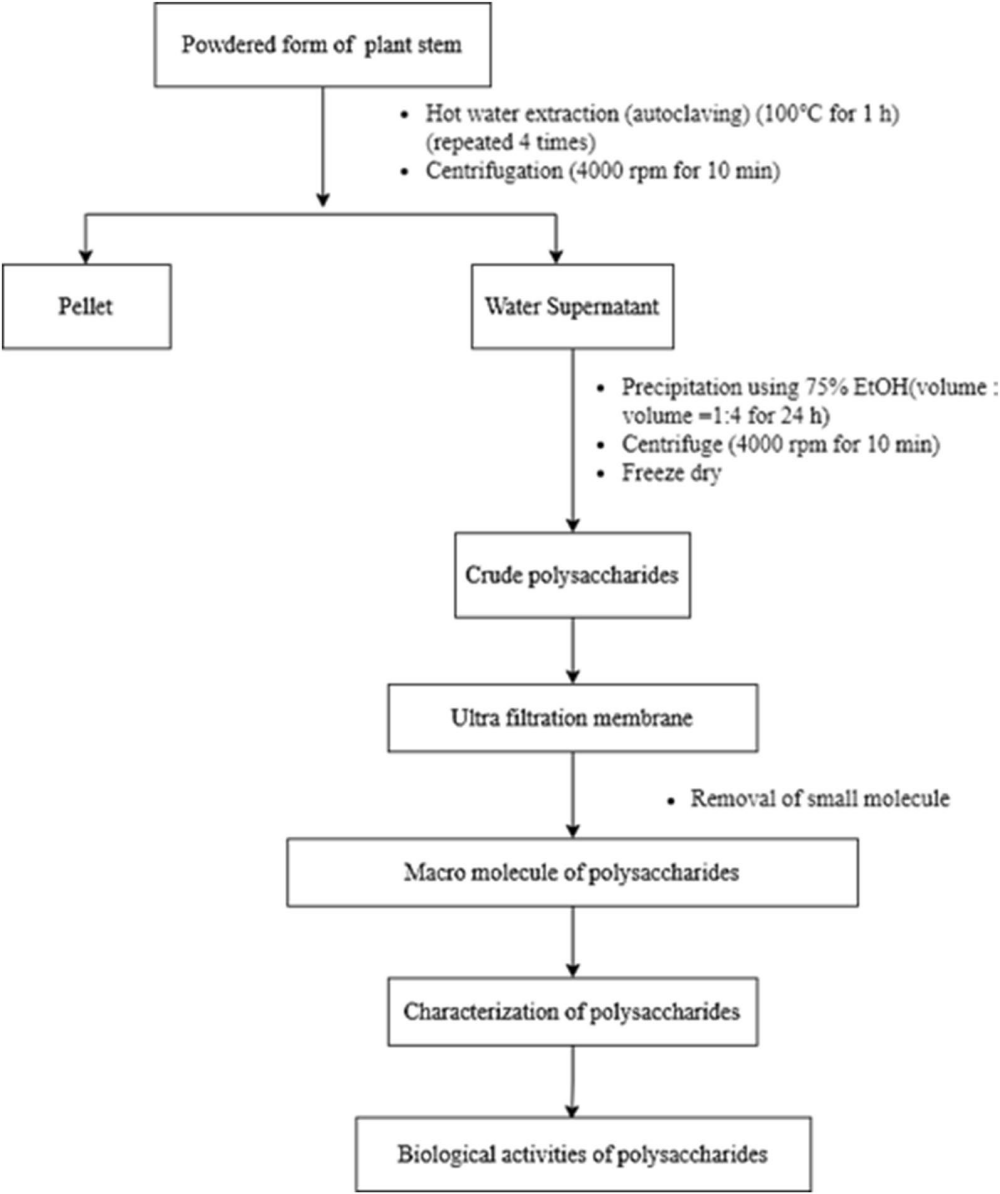
Schematic representation of the experimental process used in the study.

The complete experimental scheme for this study is shown in Fig. 2 below.

## Results

Results obtained from this study after experimental analysis are described in detail below:

### Chemical characterization of MBP

The extracted and purified MBP was tested for chemical characterization as given below.

#### FT-IR spectroscopy

**Figure 3:**
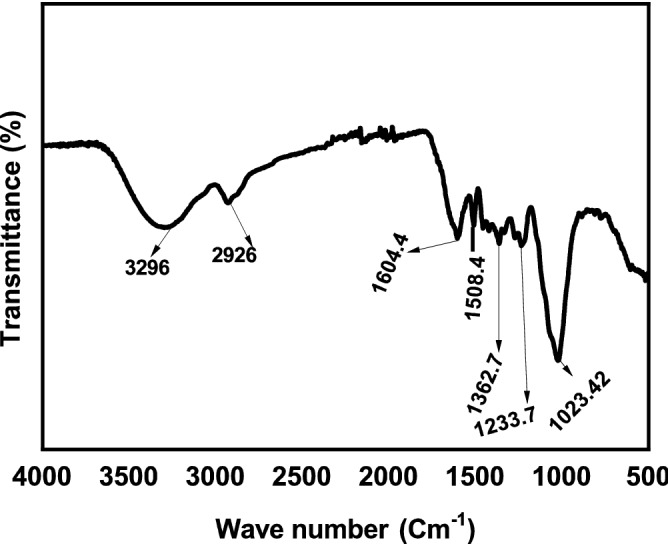
FT-IR spectra of the MBP.

One of the important methods for the prediction of structures of biological macromolecules like polysaccharides is Fourier transform infrared spectroscopy (FTIR). The FT-IR spectrum of the as-prepared material is shown in the Fig. 3. The results showed that IR spectrum of the materials had characteristic strong absorption bands at around 3296 cm^−1^ for the stretching of –OH of polysaccharides, and then weak absorption at 2926 cm^−1^ for the C–H stretching band. At 1743 cm^−1^ a weak absorption band showed the presence of carboxylic groups in the isolated polysaccharide. At 1604 cm^−1^, the stretching peaks corresponded to (C=C) carbonyl groups. The band observed at 1508 cm^−1^ showed N–O stretching, which concluded the presence of nitro compound. The two absorption peaks observed at 1362 and 1233 cm^−1^ are related to the stretching vibrations of C–H bending and C–O stretching, respectively. Moreover, an absorption band observed at 1023 cm^−1^ indicated the presence of CO–O–CO bond in the structure of material. These characteristic peaks successfully concluded the presence of polysaccharide in the observed isolated sample.

#### Scanning electron microscopy

**Figure 4:**
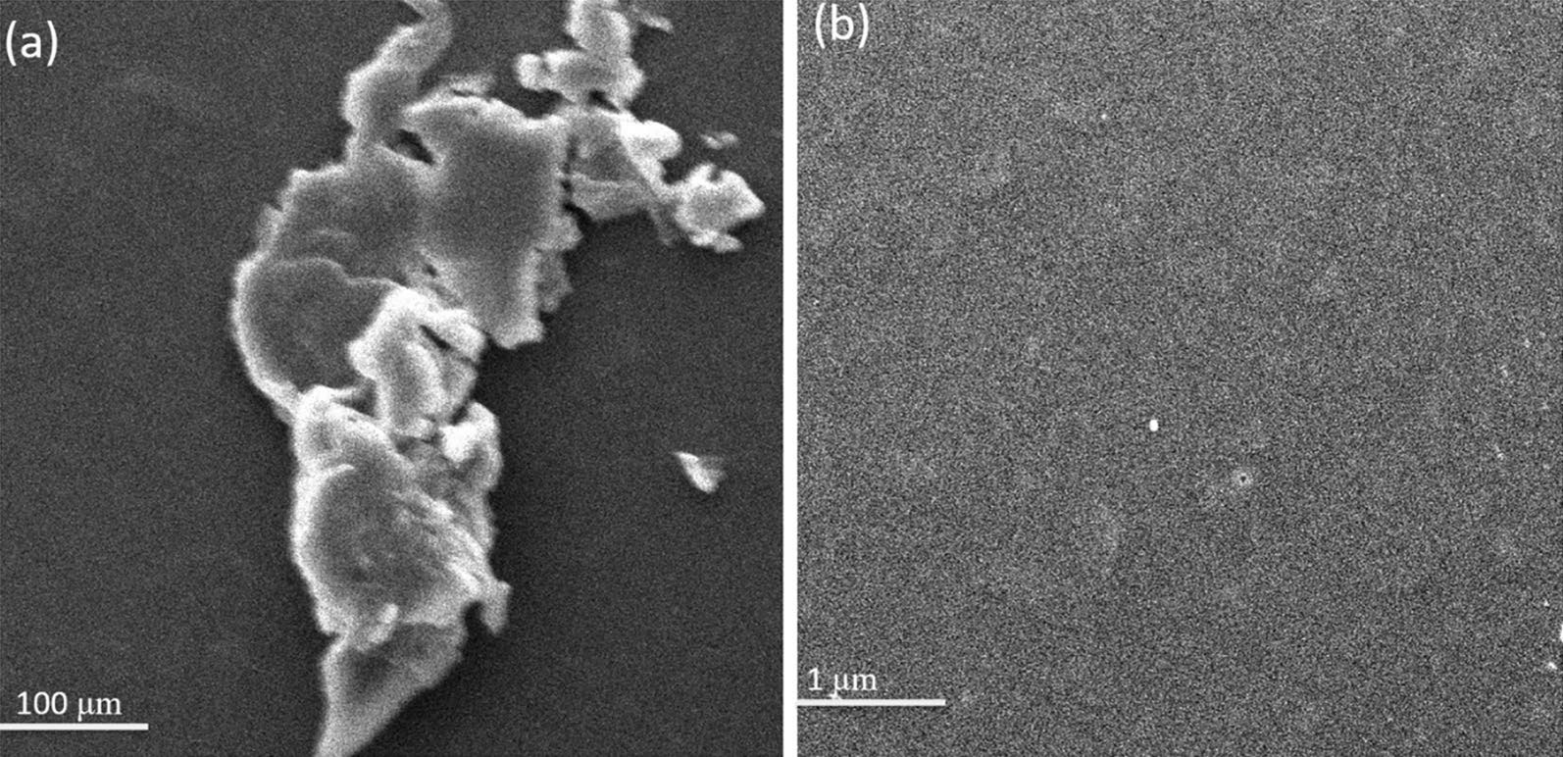
SEM micrographs of the materials. (**a**) The morphology of MBP shown at 100. (**b**) The morphology of MBP shown at 5000 magnifications.

In case of polysaccharides, complex stereo shapes exist as compared to nucleotides and proteins. In this study we used SEM for determining the morphology of the isolated polysaccharide. It was observed that the polysaccharide had a flaky shape, irregular structure and a smooth surface appearance as shown in Fig. 4. These findings confirmed that under vacuum-freeze drying conditions the isolated material was amorphous. Some string type small particles were also observed in the SEM image which showed the polysaccharide disposal; however, no difference was observed when high magnification was used.

#### XRD analysis

**Figure 5:**
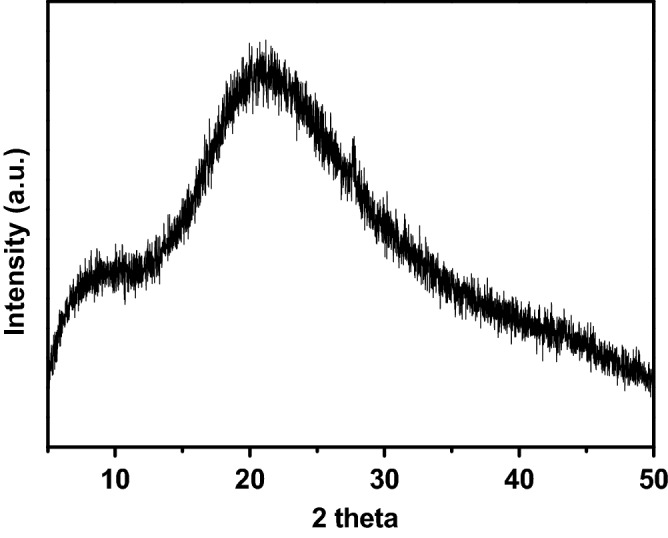
X-ray diffraction patterns of the polysaccharide.

XRD is a useful tool for deciphering polysaccharide structures, and it is also used for determining a material's crystalline structure. The XRD pattern of prepared polysaccharide was taken in the range of 5°–50° as shown in Fig. 5. The crystallinity of the sample was low, which confirms the amorphous nature of the material, with the amorphous peak regions seen at angles 20.61° and 21.26°. This was identical to the results obtained for water-soluble polysaccharides reported in literature^53^.

#### Thermal gravimetric analysis (TGA)

**Figure 6:**
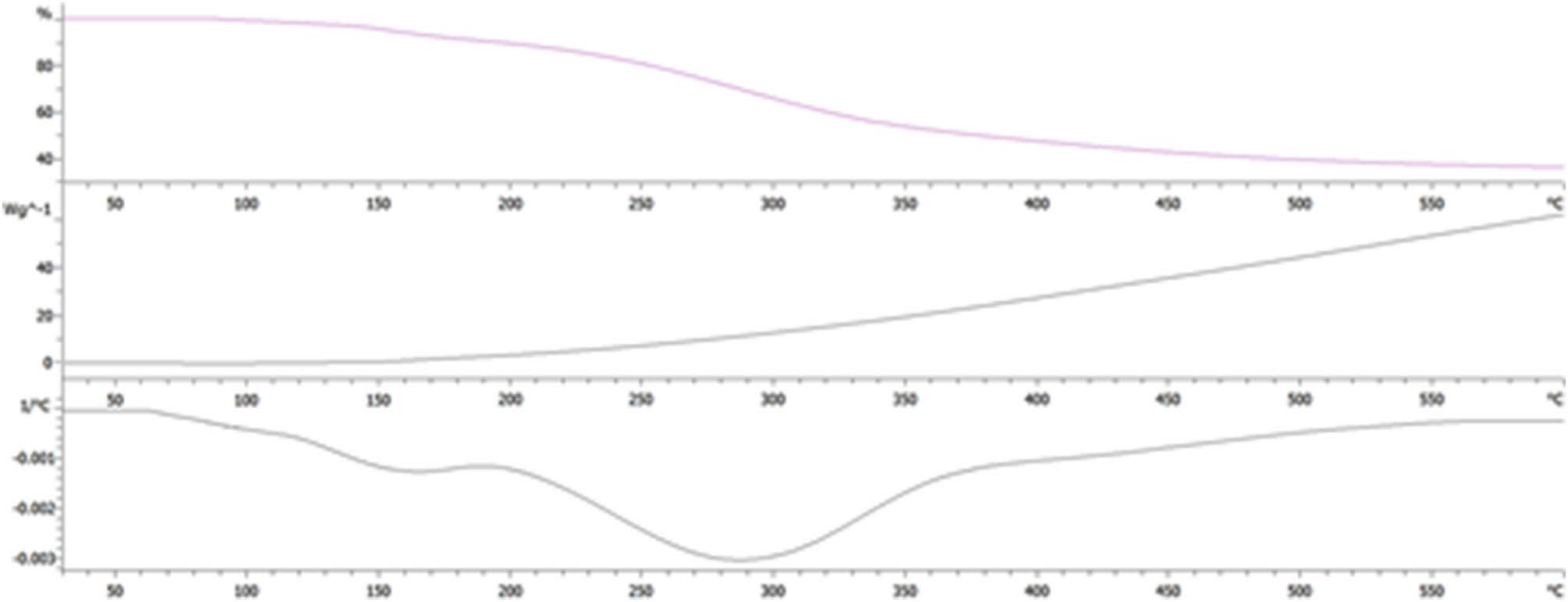
TGA analysis of the obtained polysaccharide.

Thermal analysis of prepared material was tested by using thermal gravimetric analysis (TGA), differential thermo gravimetric analysis (DTG), and differential scanning calorimetric analysis (DSC) as shown in Fig. 6. It was observed that the sample was decomposed in two stages. First stage was from 30 to 120 °C where the moisture contents from sample were removed. In second stage, from 200 to 600 °C the sample was decomposed mainly, which is due to the breakage of backbone of polysaccharide and ash formation. The results showed that at an approximately 230 °C temperature, decomposition of the material started, and it resulted in a sharp decrease of weight (42.4%) between 250 and 600 °C. The thermal analysis showed the similarity with the reported data and values are in the same range as reported by other researcher for polysaccharides^54^.

### Determination of useful biological activities

#### Antioxidant activity

The antioxidant activity of the MBP obtained in this study was measured using different parameters. The results of antioxidant potential are discussed as under.

##### Measurement of the reducing power

**Figure 7:**
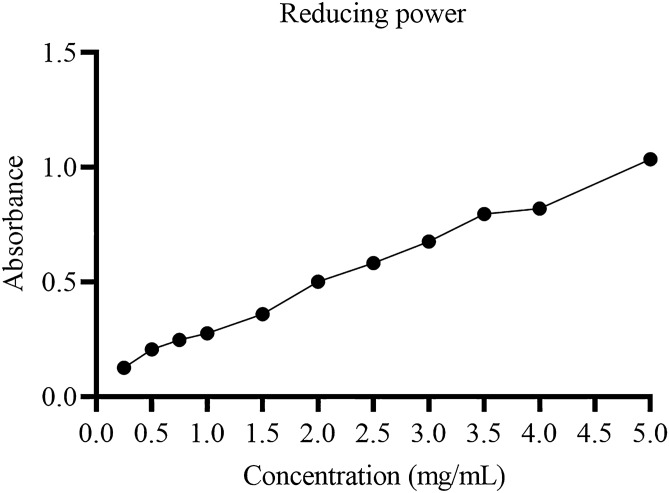
Reducing power of the MBP.

The assay which shows the reducing power of a sample relies on the fact that if antioxidants are present in a tested sample, Fe^3+^ will be reduced to Fe^2+^ upon donation of an electron. Any potential antioxidant activity is indicated by the measurement of the reducing power^55^. In this assay, the electron donating ability of antioxidants was deduced by the decrease of Fe^3+/^ferricyanide complex to the ferrous form. Spectrophotometer was used to determine the reducing power by checking absorbance of the sample at 700 nm, Fig. 7. Apparently, the reducing power of the sample increased with the increased concentrations. This study also showed that the reducing of the samples increased with the increased concentrations.

##### DPPH free radical scavenging assay

**Figure 8:**
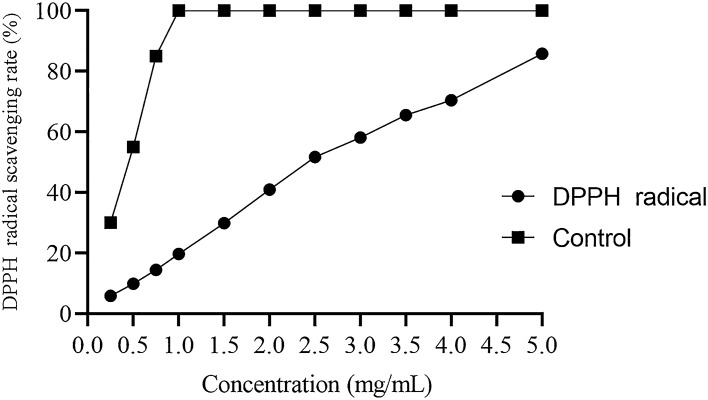
DPPH free radical scavenging activity of the MBP.

DPPH has been used for the evaluation of the free radical-scavenging capacity of natural compounds. Here, a hydrogen is donated for the formation of a stable DPPH molecule^56^. At 517 nm wavelength, this radical gives a purple color and a characteristic absorbance. The color fades when antioxidants scavenge this free radical^57^. Scavenging activities of MBP increased with the increase in sample concentration ranging from 0.5 to 5 mg/ml, and the result is shown in the Fig. 8 as compared to the control.

##### ABTS radical scavenging assay

**Figure 9:**
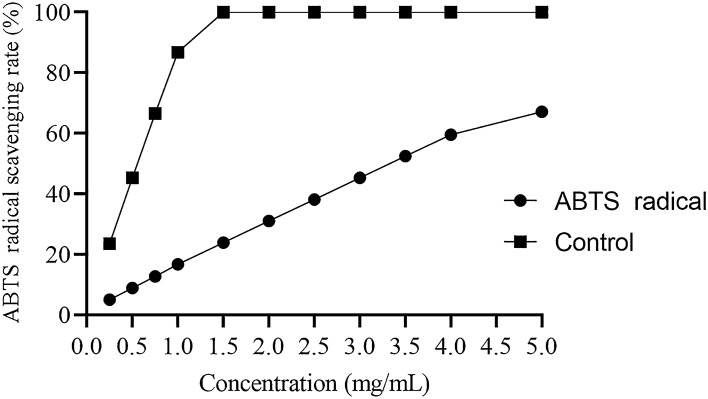
ABTS radical scavenging activity of the polysaccharide.

For measuring antioxidant activity, an assay for ABTS^+^ radical scavenging activity is an important parameter which is a simple and fast method. It can be applied for determining the antioxidant capacity of any sample containing polysaccharides^58^. The results of ABTS^+^ radical scavenging activity are shown in the Fig. 9. It was concluded that ABTS^+^ scavenging activity of our sample increased with increasing concentrations.

##### Superoxide radical scavenging assay

**Figure 10:**
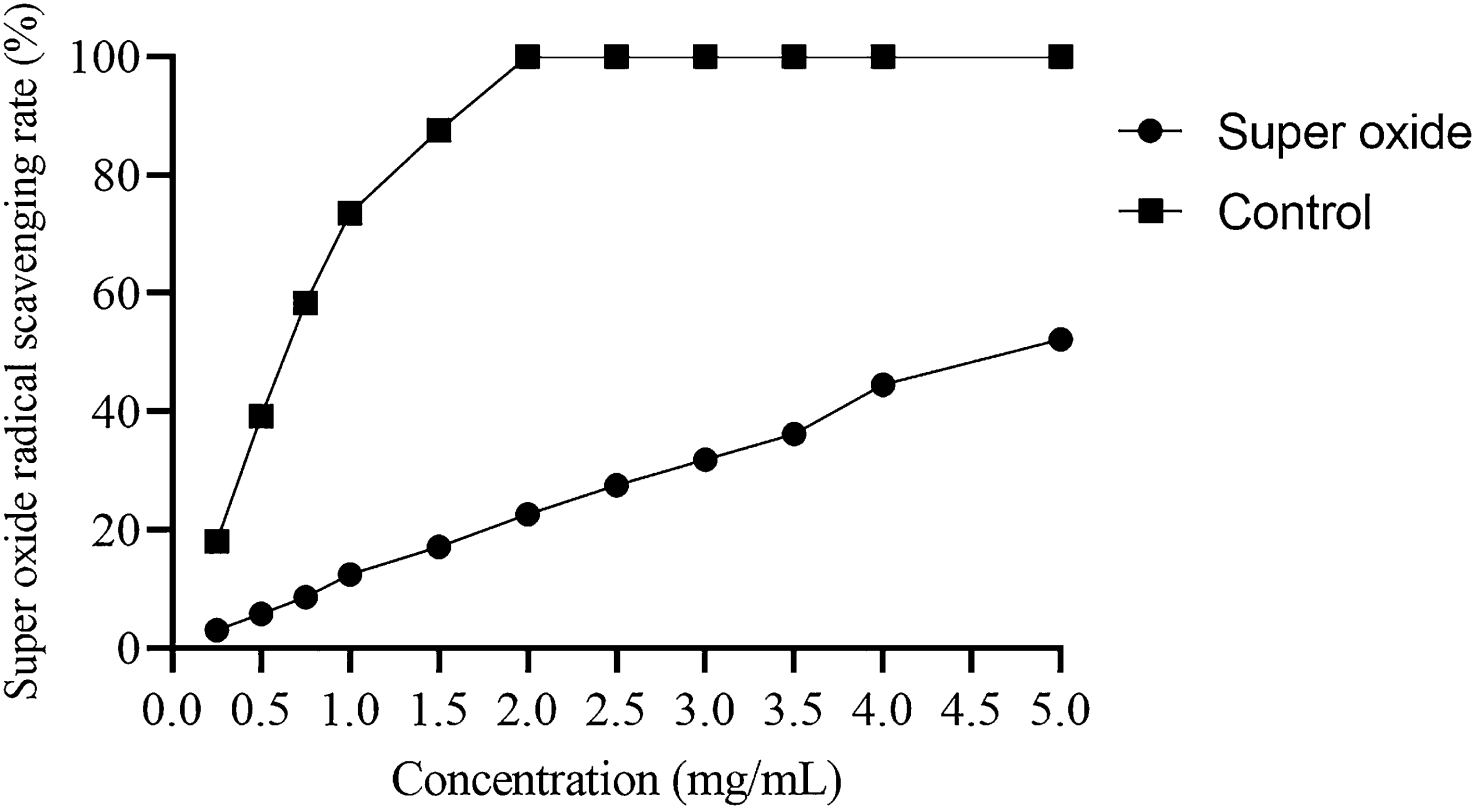
Superoxide scavenging ability of the MBP.

One of the important members among reactive oxygen species is the superoxide radical. It can act as a precursor for generation of certain other reactive oxygen species. A dismutation reaction results in the formation of H_2_O_2_ from superoxide^59^. Molecular oxygen in its ground state is reduced to superoxide radical^60^. It is considered as a weak free radical compared to other members of the ROS, but its lethality increases because of the production of other free radicals through different chemical reactions like dismutation reaction. Therefore, superoxide radical as along with its various derivations causes damage to the cells^61^. Figure 10 represents the scavenging potential of polysaccharide on the superoxide radical, and this antioxidant activity of polysaccharide is concentration dependent, when concentration increases activity is also increases.

##### Hydroxyl radical scavenging assay

**Figure 11:**
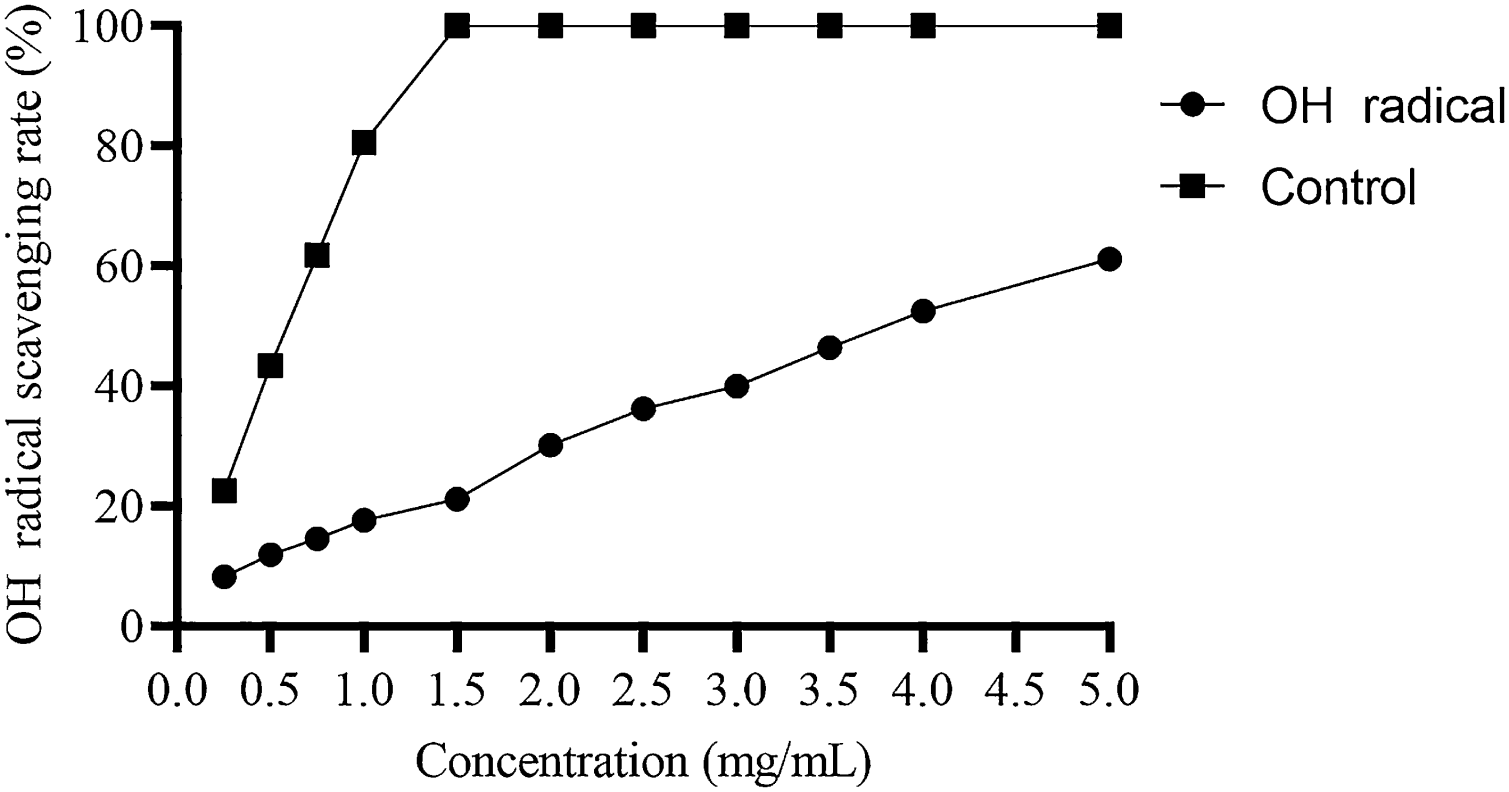
Hydroxyl scavenging ability of MBP polysaccharide.

The –OH (hydroxyl free radicals) are considered very harmful free radicals among other ROS (reactive oxygen species). These can be very lethal for different macromolecules present inside the human body, like carbohydrates, lipids, nucleic acids, as well as amino acids^62^. Therefore, removal of hydroxyl radical plays a vital role in antioxidant defense of the body. The scavenging effects of MBP on -OH (hydroxyl radical) are shown in Fig. 11. Hydroxyl radical scavenging rate of polysaccharide increased when the concentration of sample is increased. It becomes 60% when the sample concentration is 5 mg. Antioxidant activity depicted by polysaccharides is attributed to their electron or hydrogen donating ability which result in scavenging of -OH free radicals. It has been reported that clearing of hydroxyl radical is a clear indicator of antioxidant potential^63^.

##### Ferrous ion chelating activity assay

**Figure 12:**
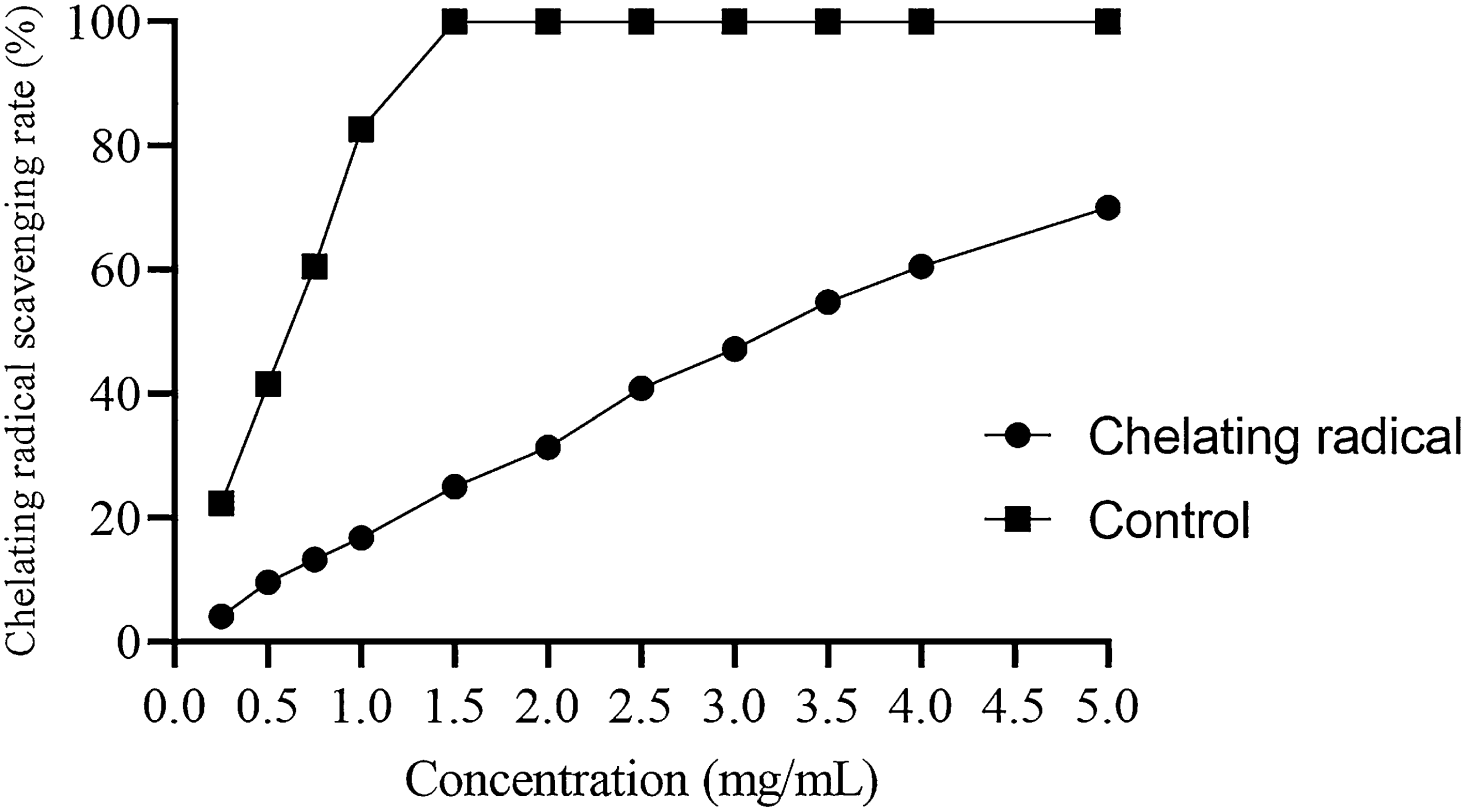
Ferrous ion chelating activity of the polysaccharide.

Antioxidant potential can be determined by measuring the iron chelating activity that affects oxidation reactions catalyzed by metals^64^. Normally, iron(II) plays a central role in oxidative damage because of the generation of free radicals through Fenton reaction. Iron(II) chelators are therefore potential effective agents against this damage of free radicals. The unavailability of iron(II) can minimize the damage caused by lipid peroxidation or other reactive oxygen species (ROS). Iron-ferrozine complexes were used for determination of iron(II) chelating activity of the samples as shown in Fig. 12. An increased concentration resulted in the increase of ferrous chelating activity of the samples. Studies have shown that deoxyribose degradation is inhibited by the molecules that cause iron chelation and make that unavailable for participation in Fenton reaction. Based on our result it can be concluded that polysaccharide extracted in this study can be used as potential antioxidant^65^.

### Antibacterial activities of MBP

The measurement of antibacterial potential of the polysaccharide obtained in this study was estimated. Given below are the results for antibacterial activity.

#### Concentration-dependent antibacterial activity of the MBP

**Figure 13:**
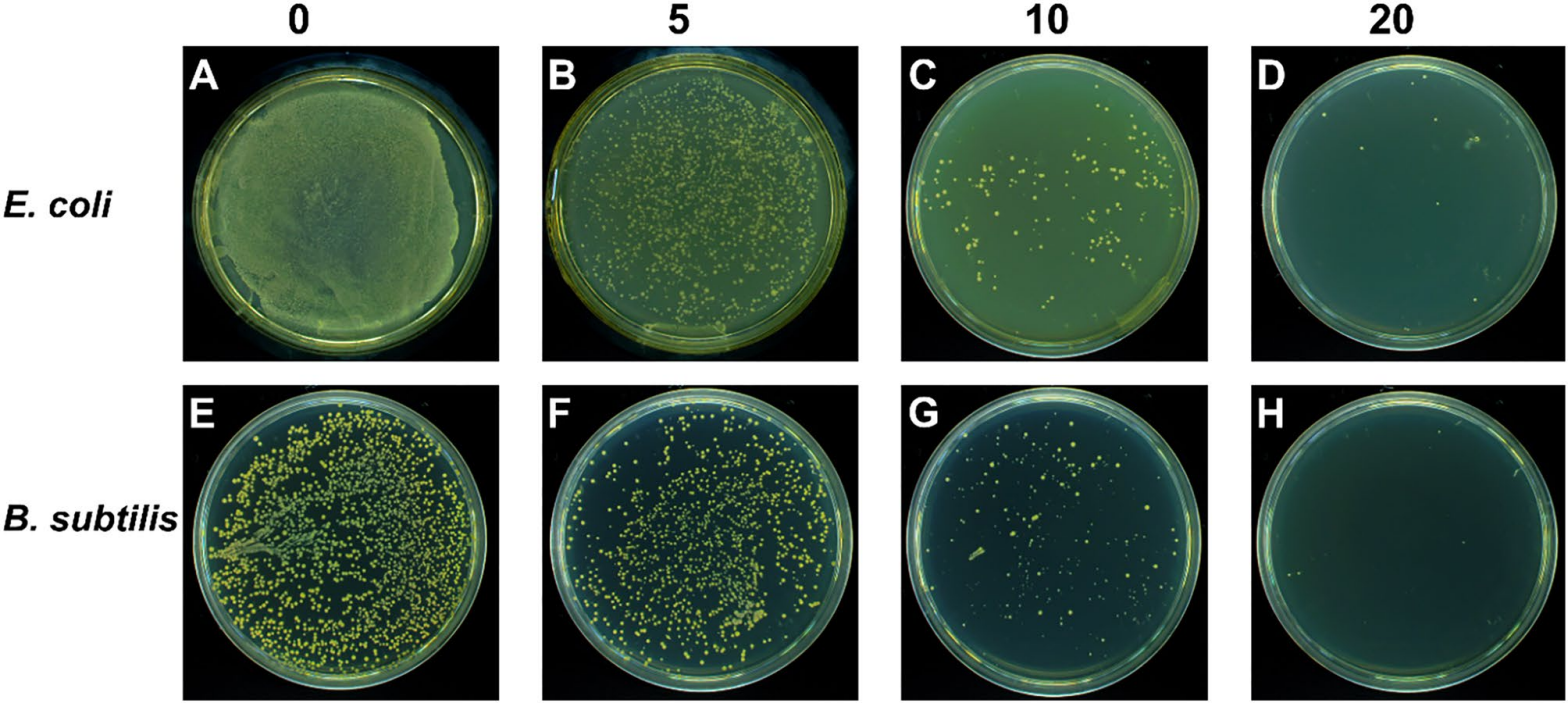
Results of *E. coli* (**A**–**D**) and *B. subtilis* (**E**–**H**) treated with various concentration at 0, 5, 10 and 20 µg/mL of MBP at 0.5, 1, 2, 4, 8, & 16, that is, different intervals of time (hours).

Measurement of cell viability and growth curve after bacterial exposure to the increased concentrations of MBP colloidal solutions measured the antibacterial activity of the MBP against *B. subtilis* and *E. coli*. Spectrophotometrically, the optical density (OD) was monitored at 600 nm for pristine, and MBP treated bacteria over various time intervals from the lag stage (where individual bacteria adapt to the environment) to the steady stage (when their growth and death rates are equivalent). Bacteria (10^7^ CFUs/ml) were recultivated and tested for measuring the bacterial count, using different MBP concentrations for 4 h. Following the treatment with different MBP concentrations, the usual photographic images of *E. coli* and *B. subtilis* colonies are shown in Fig. 13. The number of colonies decreases dramatically, as can be seen from both panels with rising MBP concentrations. The results show the antimicrobial activity depends on the administered dose of MBP.

#### Cell viability

The viability of bacterial cells after exposure to MBP in the range of 0–20 μg/mL and time interval from 0.5 to 16 h was recorded. For both gram-positive and gram-negative bacteria, MBP demonstrated an excellent antimicrobial activity.

**Figure 14:**
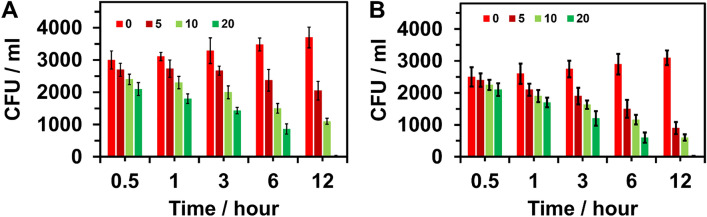
Colonies forming unit of (CFU) method to colonies (**A**) *E. coli* and (**B**) *B. subtilis* treated with various concentration at 0, 5, 10 and 20, µg/ml of MBP at 0, 3, 6, 9, and 12 different intervals of time (hour).

With increase in the concentration of MBP, the loss of growth of bacterial cells (*E. coli* and *B. subtilis*) progressively increased. At the lowest incubation time, 0.5 h with MBP concentration 5–20 μg/ml, the *E. coli* and *B. subtilis* showed the highest survival rate. The survival rate of *E. coli* and *B. subtilis* decreased by increasing the incubation time 0.5 to 6 h and increasing MBP concentration. Bacterial feasibility losses were observed at 12 h with a concentration of 20 μg/ml MBP over 100% for both bacterial strains. Figure 14 shows the effect of cell viability corresponding to the MBP concentration.

#### Optical density

**Figure 15:**
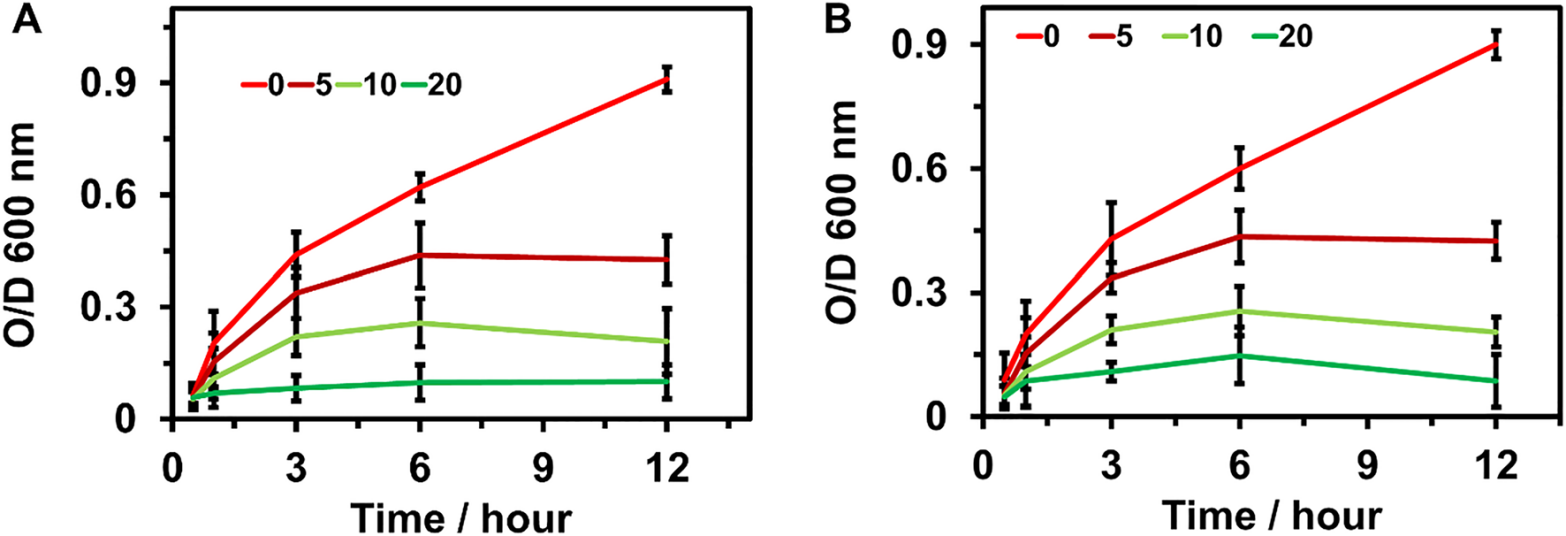
Measured OD curves of (**A**) *E. coli* and (**B**) *B. subtilis* treated with different concentrations of *Mahonia bealei* polysaccharides (MBP) at different interval times 0, 3, 6, 9, 12 h.

The bacterial regrowth curves with a second test demonstrated further that the antimicrobial activity was displayed by the MBP. Optical density cell growth curves that were incubated with various MBP concentrations at different time intervals were recorded. The bactericidal activity increased at an increasing concentration of MBP, which was consistent with the number of colonies cultivated on LB plates. There was a dose-dependent inhibition of both bacterial strains. Maximum OD was obtained from the bacterial strains at 12-h incubation without adding the MBP compound. The OD decreased by increasing the concentration of MBP to 10 µg/ml. The OD was recorded as minimal in both *E. coli* and *B. subtilis* when exposed to the strains with 10 µg/ml MBP concentration for 12 h. Results for this experiment are shown in the Fig. 15 below.

## Discussion

In this study polysaccharides from *Mahonia bealei* were extracted and purified using the hot water extraction, ethanol precipitation and ultrafiltration method. These polysaccharides were characterized using different methods and antimicrobial activity was also determined. Hot water extraction and ethanol precipitation have been reported by several studies and it is one of the cost effective methods which requires less sophistication in terms of equipment and operation used for extraction of polysaccharides from dried parts of the plants and other sources^17,66–68^. For removal of protein impurities, Sevage reagent composed of n-butanol and chloroform was used^42^. This method was used because besides the toxicity of reagent and need for the repetition of method, the rate of removal of protein is high as reported by the previous studies^67^. Ultrafiltration was performed for the separation of polysaccharides in this study. This method uses small sieves for the separation of polysaccharides keeping the structure of polysaccharides intact. Ultrafiltration and extensive dialysis resulted in the purification of polysaccharides and removal of other compounds like flavonoids, polyphenols as reported in the previous studies^43,67^.

The FT-IR spectra revealed the strong absorption band at 3296 cm^−1^ and weak absorption bands at 2926 cm^−1^ for the stretching of –OH and C–H bands respectively in consistence with the previous studies^69^. Similarly, the presence of carbonyl, carboxyl and nitro groups was concluded according to the FT-IR spectra analysis as done in the previous studies^70,71^. For determining the morphology of polysaccharides, SEM analysis was performed according to the previous studies^72^. XRD analysis also showed the results of crystalline structure of polysaccharides according to the previous studies^53^ as well as the thermal gravimetric analysis results were in consistent with the previous findings for polysaccharides from other plants^53,54^.

Among useful biological activities exhibited by the plant products, antioxidant activity is an important and useful biological activity. In this study, the DPPH radical scavenging activity increased with the increase in concentration of polysaccharide from 0.5 to 5 mg/ml concentration. The increase in antioxidant potential with the increase in quantity has been reported in the previous studies for polysaccharides from other plants as well^73^. The reducing power of MBP isolated and purified in this study was also observed to increase with the increase in concentration. This test is performed as the Fe^3+^ to Fe^2+^ reduction confirms the presence of antioxidant activity and this test is an important indicator for determining the antioxidant potential of any compound^55^. Hydroxyl ions are one of the most important free radicals that can damage macromolecules present in human body. In our study, it was observed that polysaccharide from *M. bealei* has hydroxyl radical scavenging ability. Based on previous studies and importance of hydroxyl free radicals^62^ this potential can be concluded as a useful tool for application of this polysaccharide as an antioxidant. Superoxide radical and ABTS^+^ assay further concluded the antioxidant potential of polysaccharide isolated in this study. These tests were performed for elucidating the presence of antioxidant activity in the polysaccharide previously as well^58^. During this study the antibacterial activity against gram positive and gram negative was established. It was observed that an increased concentration along with prolonged incubation time was successful in killing the representative bacterial isolates that belonged to both gram positive and gram-negative groups. Previous studies have shown that plants species that belong to genus Mahonia possess antimicrobial potential^27,74^. However, to the best of our knowledge no study has been conducted on exploration of antimicrobial potential from purified polysaccharides of *M. bealei*; therefore, this study will help in the future exploration of antimicrobial potential from polysaccharides of *M. bealei*.

*Mahonia aquifolium*, another important and related specie of *M. bealei* was explored for therapeutic potential of its polysaccharides^75,76^. The polysaccharides isolated using the stem of *M. aquifolium* was found a potent inducer for the production of IL-8, an important interleukin of the human immune system^75^. Antioxidant activity was reported by the eleven polysaccharides extracted from different plants including *M. aquifolium* in another study^76^. In this study polysaccharide was extracted, isolated, and purified using the stem of *M. bealei* for the first time. As previous studies have shown that polysaccharides are one of the very important biomacromolecules that depend on their structure for their activity; therefore, further studies should be done to determine the potential relationships of their structures and biological activities. However, as other compounds from this plant have been isolated and studied, polysaccharides have been underexplored, the gap that we have endeavored to fill through this study. This study will provide a better insight for researchers for future studies on polysaccharides of *M. bealei*.

## Conclusion

*M. bealei* is an important member of the genus Mahonia and has been used extensively in the TCM throughout the history. Different classes of compounds like flavonoids etc. have been isolated, purified, and their useful biological activities have been explored. The extraction, purification, characterization, and exploration of polysaccharides from this plant have been underexplored. In this study the extraction and ultrafiltration procedures yielded polysaccharide MBP as determined by FT-IR, SEM, XRD, and TGA analysis. Antioxidant and antimicrobial potentials of MBP were explored and determined. Our literature survey shows that the polysaccharides from *M. bealei* have not been explored and this study will provide an insight for future studies to be conducted and exploring the potential therapeutic effects of polysaccharides from different parts of *M. bealei*.
